# Supplementary material for: Role of Monomer/Tetramer Equilibrium of Rod Visual Arrestin in the Interaction with Phosphorylated Rhodopsin
Source: Int J Mol Sci. 2023 Mar 4;24(5):4963. doi: 10.3390/ijms24054963 (PMC10003454; doi:10.3390/ijms24054963)
Supplement: Supplementary file 1 [file ijms-24-04963-s001.zip › imamoto_et_al_supplementary.pdf]

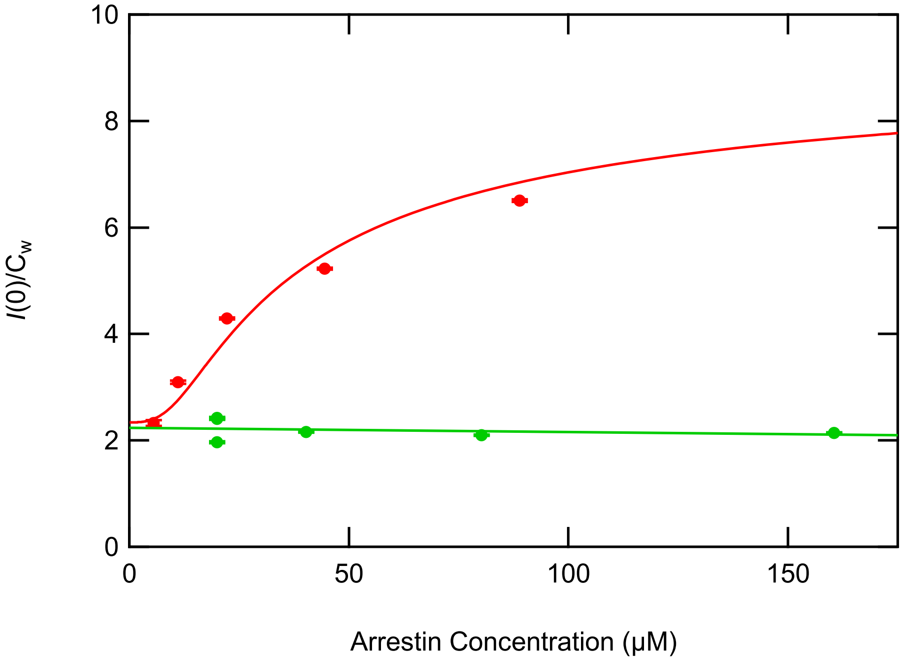

Figure S1: Concentration dependence of $I(0)/C_w$ for arrestin (red) and ovalbumin (green). $I(0)/C_w$ for ovalbumin (green) was fitted by linear line, whereas that of arrestin was fitted with the curve generated by Eq. 1 to estimate K_{Arr}. $I(0)/C_W$ for arrestin monomer was estimated by using $I(0)/C_W$ for ovalbumin and molecular weights of arrestin (45 K) and ovalbumin (43 K).

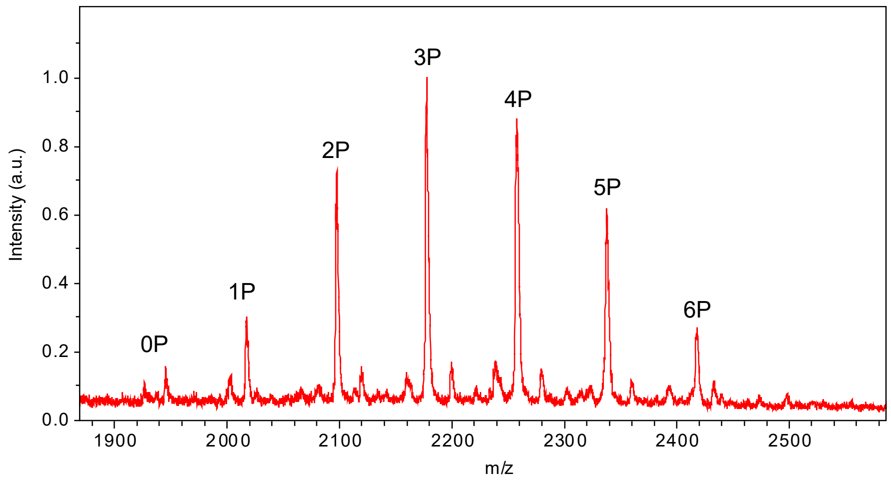

Figure S2: MALDI TOF MS of the C-terminal fragment of phosphorylated rhodopsin.
